# Supplementary material for: Multi-ancestry and multi-trait genome-wide association meta-analyses inform clinical risk prediction for systemic lupus erythematosus
Source: Nat Commun. 2023 Feb 7;14:668. doi: 10.1038/s41467-023-36306-5 (PMC9905560; doi:10.1038/s41467-023-36306-5)

Supplementary Information for

Multi-ancestry and multi-trait genome-wide association meta-analyses inform clinical risk prediction for systemic lupus erythematosus

Authors:
Chachrit Khunsriraksakul[1,2], Qinmengge Li[3], Havell Markus[1,2], Matthew T. Patrick[3], Renan Sauteraud[4], Daniel McGuire[4], Xingyan Wang[4], Chen Wang[1], Lida Wang[4], Siyuan Chen[4], Ganesh Shenoy[5], Bingshan Li[6], Xue Zhong[7], Nancy J. Olsen[8], Laura Carrel[9], Lam C. Tsoi[3,#], Bibo Jiang[4,#], Dajiang J. Liu[1,2,4,*,#]

1 Program in Bioinformatics and Genomics; Pennsylvania State University College of Medicine; Hershey, Pennsylvania, 17033; USA.
2 Institute for Personalized Medicine; Pennsylvania State University College of Medicine; Hershey, Pennsylvania, 17033; USA.
3 Department of Dermatology; University of Michigan Medical School; Ann Arbor, Michigan, 48109; USA.
4 Department of Public Health Sciences; Pennsylvania State University College of Medicine; Hershey, Pennsylvania, 17033; USA.
5 Department of Neurosurgery; Pennsylvania State University College of Medicine; Hershey, Pennsylvania, 17033; USA.
6 Department of Molecular Physiology & Biophysics; Vanderbilt University; Nashville, Tennessee, 37235; USA.
7 Department of Medicine, Division of Genetic Medicine; Vanderbilt University Medical Center; Nashville, Tennessee, 37232; USA.
8 Department of Medicine; Pennsylvania State University College of Medicine; Hershey, Pennsylvania, 17033; USA.
9 Department of Biochemistry and Molecular Biology; Pennsylvania State University College of Medicine; Hershey, Pennsylvania, 17033; USA.

#: These authors jointly supervised the work.
Manuscript correspondence should be addressed to:
Dajiang J. Liu: dajiang.liu@psu.edu

This file includes:

Supplementary Tables 1 to 5
Supplementary Figures 1 to 8

Supplementary Table 1: Multi-ancestry GWAS results for 14 autoimmune diseases across 16 novel and replicable loci identified in multi-ancestry and multi-trait SLE GWAS. Three loci are already significant in multi-ancestry GWAS of SLE only (highlighted in green box). Blue box represents the trait (other than SLE) with the smallest P value for each locus. Importantly, rheumatoid arthritis (RA) contributes the most to the identification of novel loci in SLE, having the smallest P values for 8 out of 16 loci in comparison to other autoimmune diseases, which is possibly due to its large sample sizes. Two-sided P value associated with each variant is calculated according to the Chi-squared test statistic with 1 degree of freedom.

rsID	Variant ID	SLE	ATD	CD	CEL	MS	PBC	RA	SJO	SSC	T1D	UC
rs6662618	1_92935411_T_G_b37	1.48×10^{-10}	9.27×10^{-1}	2.43×10^{-3}	8.21×10^{-1}	8.39×10^{-7}	9.31×10^{-1}	5.48×10^{-5}	2.01×10^{-2}	3.07×10^{-2}	2.91×10^{-4}	1.64×10^{-1}
rs2453044	1_120508524_A_G_b37	4.10×10^{-6}	4.49×10^{-1}	1.03×10^{-4}	8.53×10^{-1}	8.94×10^{-1}	6.17×10^{-2}	1.55×10^{-1}	3.69×10^{-1}	9.58×10^{-1}	4.94×10^{-7}	6.12×10^{-1}
rs12992553	2_70360262_A_G_b37	4.59×10^{-6}	7.35×10^{-1}	6.65×10^{-1}	2.34×10^{-1}	5.13×10^{-1}	1.43×10^{-2}	1.01×10^{-2}	2.85×10^{-1}	1.14×10^{-3}	1.38×10^{-1}	9.64×10^{-1}
rs13014122	2_135050622_G_A_b37	2.03×10^{-8}	4.28×10^{-1}	5.17×10^{-1}	4.27×10^{-1}	1.81×10^{-1}	4.18×10^{-2}	2.53×10^{-2}	9.03×10^{-1}	1.38×10^{-1}	3.31×10^{-1}	9.03×10^{-1}
rs299629	3_12576846_A_G_b37	6.08×10^{-5}	4.41×10^{-1}	9.56×10^{-3}	9.11×10^{-1}	2.91×10^{-1}	2.18×10^{-2}	7.30×10^{-6}	4.05×10^{-2}	4.92×10^{-4}	8.56×10^{-4}	9.71×10^{-1}
rs12490565	3_121553719_G_A_b37	1.76×10^{-7}	7.40×10^{-1}	2.47×10^{-1}	2.96×10^{-1}	6.35×10^{-2}	1.77×10^{-2}	1.20×10^{-3}	6.40×10^{-1}	2.05×10^{-3}	2.34×10^{-1}	7.25×10^{-2}
rs4697651	4_10721433_C_T_b37	4.01×10^{-6}	1.14×10^{-2}	2.03×10^{-1}	1.64×10^{-1}	2.62×10^{-1}	7.37×10^{-1}	1.46×10^{-6}	8.02×10^{-1}	7.68×10^{-1}	1.67×10^{-2}	4.24×10^{-1}
rs2288786	5_102600754_G_A_b37	8.19×10^{-5}	3.62×10^{-1}	1.45×10^{-2}	1.54×10^{-1}	2.51×10^{-1}	1.27×10^{-7}	1.66×10^{-8}	4.97×10^{-2}	6.73×10^{-1}	1.48×10^{-2}	1.59×10^{-1}
rs12529514	6_14096658_T_C_b37	1.70×10^{-5}	9.32×10^{-2}	1.85×10^{-1}	9.06×10^{-1}	5.85×10^{-1}	3.46×10^{-2}	4.52×10^{-11}	2.21×10^{-1}	2.66×10^{-1}	1.62×10^{-1}	8.28×10^{-1}
rs6939565	6_130194204_C_T_b37	5.38×10^{-5}	4.13×10^{-1}	1.05×10^{-1}	9.39×10^{-1}	4.22×10^{-1}	4.22×10^{-4}	6.75×10^{-3}	5.19×10^{-2}	1.57×10^{-1}	1.95×10^{-3}	4.15×10^{-2}
rs9494331	6_136006301_G_A_b37	1.49×10^{-6}	1.36×10^{-1}	6.23×10^{-1}	6.53×10^{-1}	5.12×10^{-2}	2.66×10^{-2}	2.33×10^{-1}	1.50×10^{-1}	3.07×10^{-1}	6.37×10^{-3}	1.73×10^{-1}
rs3761847	9_123690239_G_A_b37	2.32×10^{-6}	1.72×10^{-1}	7.23×10^{-1}	4.16×10^{-3}	9.11×10^{-1}	4.08×10^{-1}	3.59×10^{-8}	6.88×10^{-5}	4.10×10^{-2}	2.39×10^{-3}	2.97×10^{-1}
rs6602588	10_12487996_G_A_b37	4.35×10^{-8}	2.71×10^{-2}	1.15×10^{-1}	1.25×10^{-1}	3.14×10^{-1}	4.22×10^{-1}	9.75×10^{-1}	5.81×10^{-1}	4.99×10^{-3}	2.30×10^{-1}	7.77×10^{-1}
rs516124	11_64128423_G_T_b37	1.46×10^{-6}	6.06×10^{-2}	1.48×10^{-5}	4.34×10^{-1}	7.11×10^{-1}	3.75×10^{-10}	3.38×10^{-10}	2.51×10^{-1}	1.19×10^{-2}	1.27×10^{-6}	7.38×10^{-1}
rs199533	17_44828931_G_A_b37	3.28×10^{-6}	7.80×10^{-1}	3.88×10^{-2}	4.27×10^{-1}	9.35×10^{-1}	2.20×10^{-4}	7.12×10^{-1}	6.38×10^{-4}	3.08×10^{-2}	1.70×10^{-5}	4.97×10^{-1}
rs1535271	20_57734753_G_A_b37	9.14×10^{-7}	7.22×10^{-1}	2.91×10^{-2}	7.72×10^{-3}	6.09×10^{-1}	4.69×10^{-5}	2.46×10^{-3}	1.23×10^{-1}	1.30×10^{-1}	9.09×10^{-2}	3.31×10^{-3}

Disease name abbreviations: AN = ankylosing spondylitis, ATD = autoimmune thyroid disease, CD = Crohn's disease, CEL = celiac disease, MS = multiple sclerosis, PBC = primary biliary cirrhosis, PSOA = psoriatic arthritis, RA = rheumatoid arthritis, SJO = Sjogren's syndrome, SLE = systemic lupus erythematosus, SSC = systemic sclerosis, T1D = type 1 diabetes, UC = ulcerative colitis, VIT = vitiligo.

Supplementary Table 2: Number of variants in each PRS model. The model is trained using multi-ancestry and multi-trait (MAMT) SLE GWAS data and LD reference panel from 1000 Genomes project (European ancestry). The p-value thresholds used for the P+T method are listed in brackets.

**Model**	**Number of variants**
P+T [5×10^{-8}]	206
P+T [5×10^{-7}]	259
P+T [1×10^{-6}]	283
P+T [5×10^{-6}]	349
P+T [5×10^{-5}]	524
P+T [5×10^{-4}]	952
P+T [5×10^{-3}]	2,954
P+T [1×10^{-2}]	4,674
P+T [5×10^{-2}]	15,696
P+T [1×10^{-1}]	26,866
P+T [2×10^{-1}]	45,234
P+T [5×10^{-1}]	84,273
P+T [1×10^{0}]	119,342
SBayesR	896,086
GCTA	893,016
Ldpred-Inf	896,086
PUMAS	330
Ldpred-funct	519,899
SDPR	896,086
PRS-CS-auto	877,453
LASSOSUM	8,657

Supplementary Table 3: ICD codes used to define SLE cases and exclusion criteria.

Disease	ICD codes
Systemic lupus erythematosus (SLE)	**ICD9:** 710.0<br>**ICD10:** M32.8, M32.9, M32.10, M32.11, M32.12, M32.13, M32.14, M32.15, M32.19
Systemic sclerosis (SSC)	**ICD9:** 710.1<br>**ICD10:** M34, M34.0, M34.2, M34.8, M34.9
Dermatomyositis (DM)	**ICD9:** 710.3<br>**ICD10:** M33, M33.0, M33.1, M33.9
Unspecified connective tissue disease (UCTD)	**ICD9:** 710.9<br>**ICD10:** M35.9

Supplementary Table 4: Algorithms used to define SLE cases in electronic health records according to Barnado et al., 2017.

**Algorithm**	**Description**
Def1<br>(Least stringent)	≥ 1 SLE ICD code counts
Def6<br>(Intermediate)	≥ 2 SLE ICD code counts & Excluding SSC and DM
Def12<br>(Most stringent)	≥ 4 SLE ICD code counts & Excluding SSC and DM & ANA ≥ 1:160

Abbreviations: SSC = systemic sclerosis, DM = dermatomyositis

Supplementary Table 5: Demographic characteristics of patients with SLE in MGI and BioVU according to different algorithms used to define SLE.

**Variable**	***MGI SLE cases***			***BioVU SLE cases***		
	**Def1**	**Def6**	**Def12**	**Def1**	**Def6**	**Def12**
N total (N, %)	359 (100%)	164 (100%)	37 (100%)	749 (100%)	385 (100%)	173 (100%)
Females	312 (86.9%)	142 (86.6%)	33 (89.2%)	633 (84.5%)	342 (88.8%)	152 (87.9%)
Males	47 (13.1%)	22 (13.4%)	4 (10.8%)	116 (15.5%)	43 (11.2%)	21 (12.1%)
ANA status (N, %)	324 (100%)	139 (100%)	37 (100%)	465 (100%)	262 (100%)	173 (100%)
ANA (+)	226 (69.8%)	90 (64.7%)	37 (100%)	386 (83.0%)	224 (85.5%)	173 (100%)
ANA (-)	98 (30.2%)	49 (35.3%)	0 (0%)	79 (17.0%)	38 (14.5%)	0 (0%)
anti-dsDNA status (N, %)	310 (100%)	141 (100%)	35 (100%)	416 (100%)	264 (100%)	154 (100%)
anti-dsDNA (+)	41 (13.2%)	19 (13.5%)	9 (25.7%)	105 (25.2%)	75 (28.4%)	49 (31.8%)
Anti-dsDNA (-)	269 (86.8%)	122 (86.5%)	26 (74.3%)	311 (74.8%)	189 (71.6%)	105 (68.2%)
UCTD diagnosis	116 (32.3%)	44 (26.8%)	14 (37.8%)	70 (9.3%)	40 (10.4%)	22 (12.7%)

Abbreviation: SLE = systemic lupus erythematosus, UCTD = undifferentiated connective tissue disease

Supplementary Figure 1: Overview of the study design and workflow. We first conduct multi-ancestry fixed effect meta-analysis (MA) of SLE GWAS studies. We also conduct a multi-ancestry & multi-trait meta-analysis (MAMT) of SLE and SLE-related GWAS studies. Specifically, we first determine which autoimmune diseases are genetically correlated with SLE via LDSC using GWAS of European ancestry. We then perform multi-trait analysis of SLE and SLE-related traits within each ancestry via MTAG. Then, we perform multi-ancestry fixed effect meta-analysis of multi-trait SLE GWAS. For MAMT, we assess the replicability of identified association signals. We also perform transcriptome-wide association study (TWAS), and use the results to conduct cell type enrichment (CTE) analysis to identify immune cell types that are enriched with TWAS signals and computational drug repurposing (CDR) analysis to identify drugs that we may repurpose to treat SLE. We then calculate polygenic risk score for SLE using nine PRS methods, including pruning and thresholding (P+T), SBayesR, SBLUP, SDPR, LDpred-Inf, LDpred-funct, PUMAS, PRS-CS-auto, and LASSOSUM. In total, 27 candidate PRS models were derived from three SLE GWAS summary statistics and nine PRS methods. Using two independent biobanks with electronic medical record, i.e., Vanderbilt BioVU and Michigan Genomics Initiative, we demonstrated that combining PRS and conventional lab tests (antinuclear antibody and anti-double stranded DNA tests) can improve diagnostic accuracy of SLE.

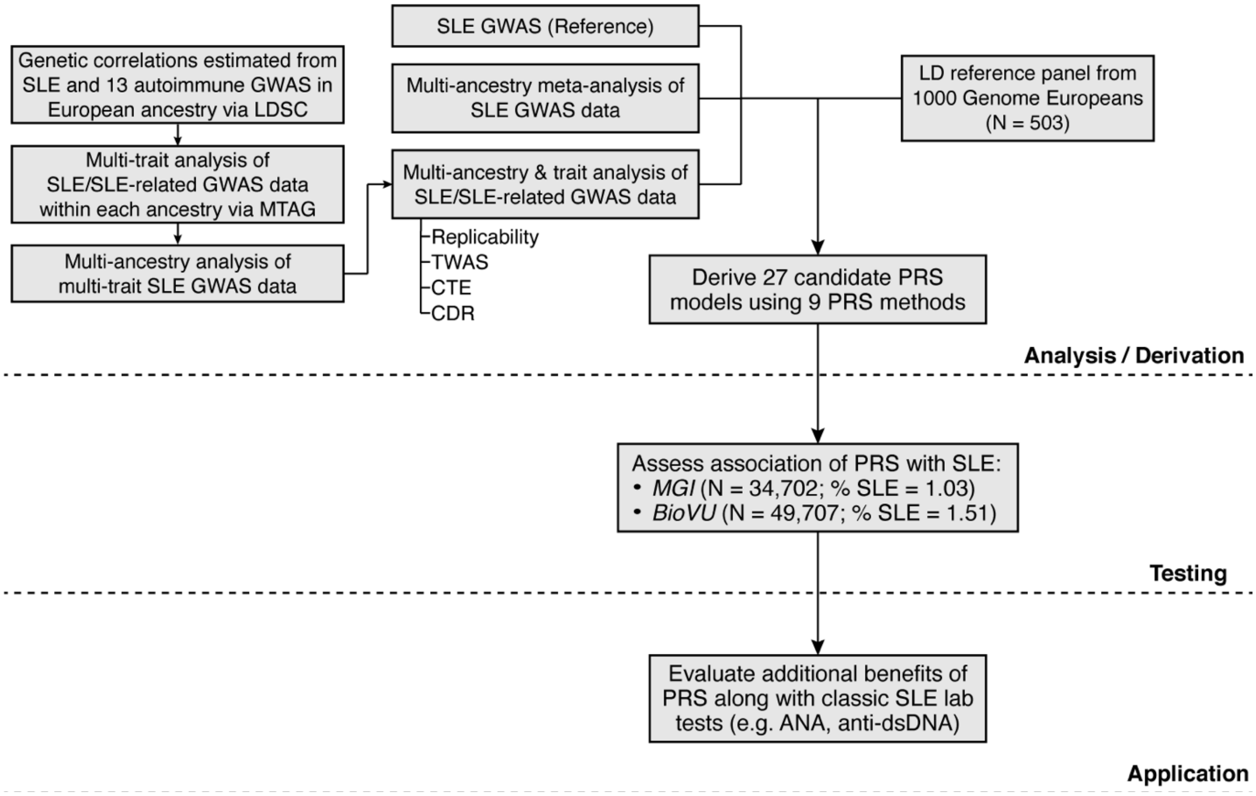

Supplementary Figure 2: Effective sample sizes across 14 autoimmune diseases. We calculate effective sample size (N_{eff}) according to the formula $2/(1/N_{cases} + 1/N_{controls})$, which is a more informative measure of the sample size for datasets with unbalanced number of cases and controls. Colors represent sample ancestries.

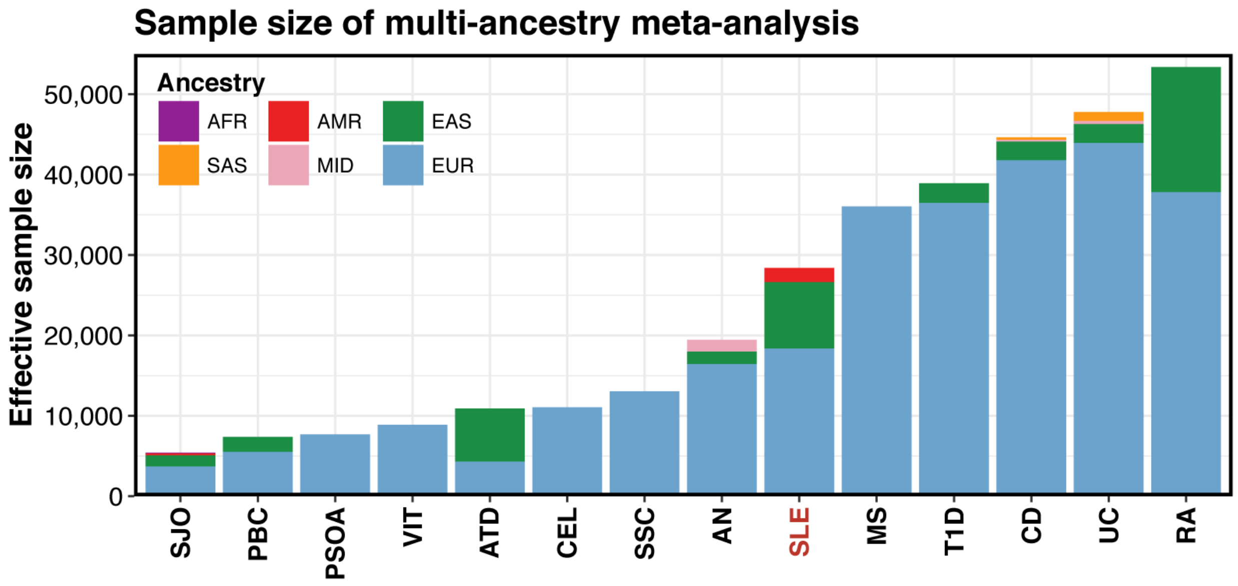

Abbreviations: AFR = African, AMR = Admixed American, EAS = East Asian, EUR = European, MID = Middle Eastern, SAS = South Asian

Supplementary Figure 3: QQ plot for multi-ancestry and multi-trait meta-analysis. Genomic control inflation factor is 1.03. Two-sided P value associated with each variant is calculated according to the Chi-squared test statistic with 1 degree of freedom.

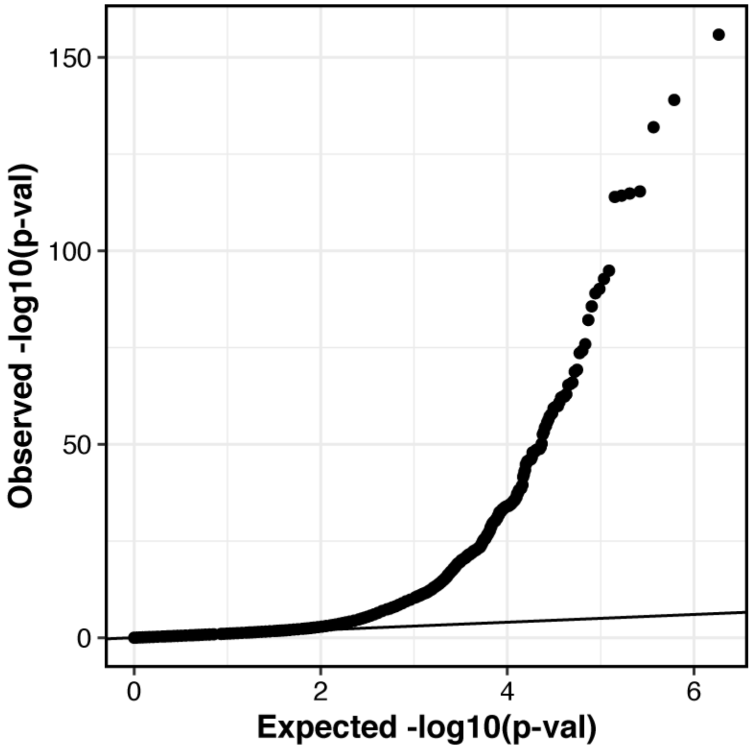

Supplementary Figure 4: Cohort-level Z scores for sentinel variants at 27 novel loci. (**A**) RATES identifies 16 novel loci that are replicable (posterior probability of replicability > 0.90). (**B**) RATES detects 11 novel loci that are not replicable (posterior probability of replicability ≤0.90). Each dot represents the Z-score from a study. The sizes of the dots represent the sample size of the participating cohorts, while the colors represent the ancestry of the cohorts. "X" represents the meta-analysis effect sizes. Minima and maxima values (excluding outliers) are represented by the lower- and upper-bound of the whiskers. Median value is represented by the bold line in the middle of the box. First and third quartiles are represented by the lower- and upper-bound of the box. A notable feature of un-replicable signals is that some small participating studies contribute disproportionally large Z-scores, indicating that they may be an outlier.

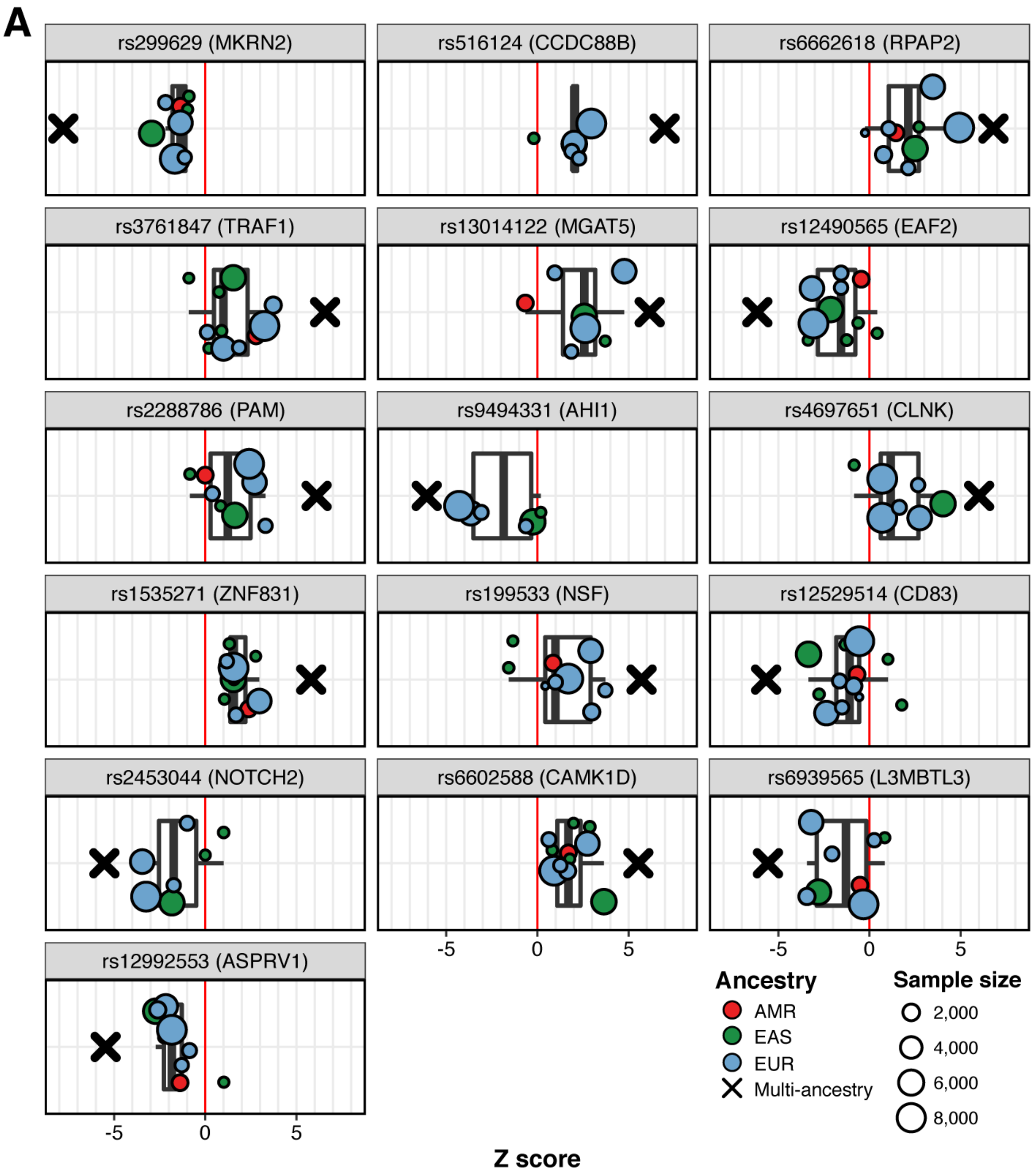

B

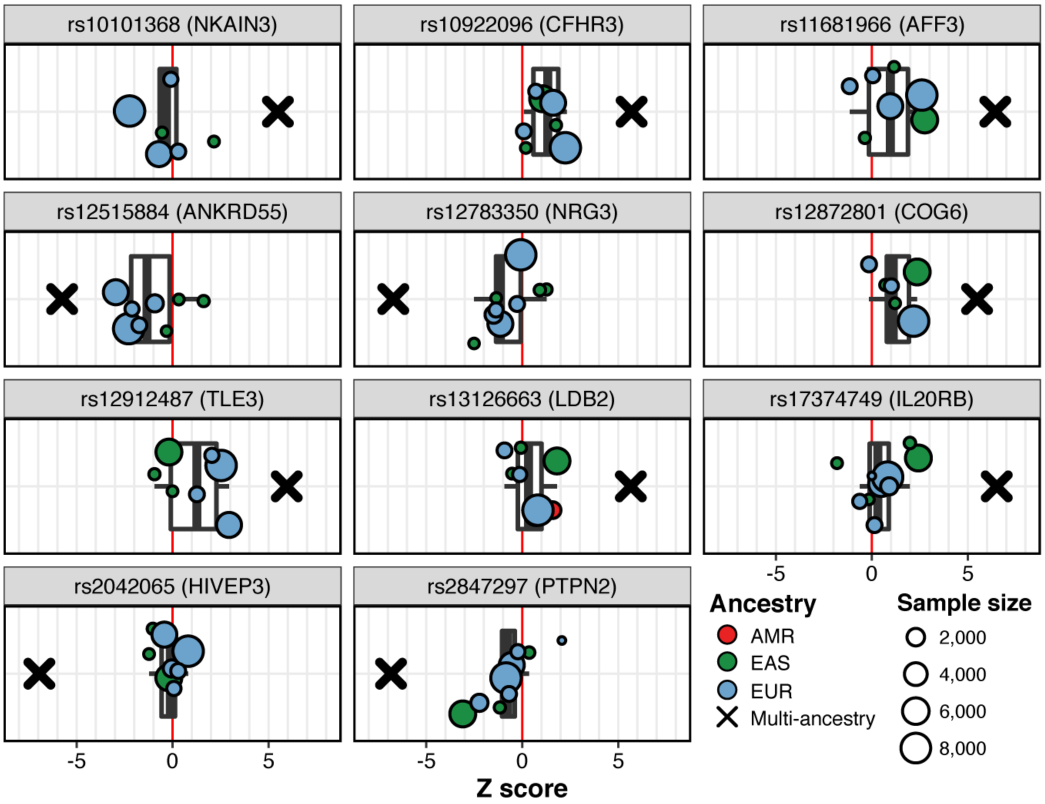
rs10101368 (NKAIN3)
rs10922096 (CFHR3)
rs11681966 (AFF3)
rs12515884 (ANKRD55)
rs12783350 (NRG3)
rs12872801 (COG6)
rs12912487 (TLE3)
rs13126663 (LDB2)
rs17374749 (IL20RB)
rs2042065 (HIVEP3)
rs2847297 (PTPN2)
-5
0
5
Z score
Ancestry
AMR
EAS
EUR
Multi-ancestry
Sample size
2,000
4,000
6,000
8,000

Supplementary Figure 5: Concordance of effect size (beta) between biobank datasets and GWAS discovery cohorts. X-axis represents beta from the GWAS discovery cohorts, while Y-axis indicates beta from the biobank datasets (MGI & BioVU). Here, only the significant variants (P value $< 5 \times 10^{-8}$) from the discovery cohorts are illustrated. Blue dots represent variants with P value $< 5 \times 10^{-8}$ in biobank datasets. We defined SLE cases in biobank datasets using 3 different algorithms (Def1, Def6, Def12) according to **Supplementary Table 4**. R and p refer to Pearson's correlation and its associated two-sided P value respectively. The red line represents the relationship between genetic effects in the discovery GWAS, and in BioVU and MGI biobanks. The shaded area surrounding the line represents the 95%-confidence band.

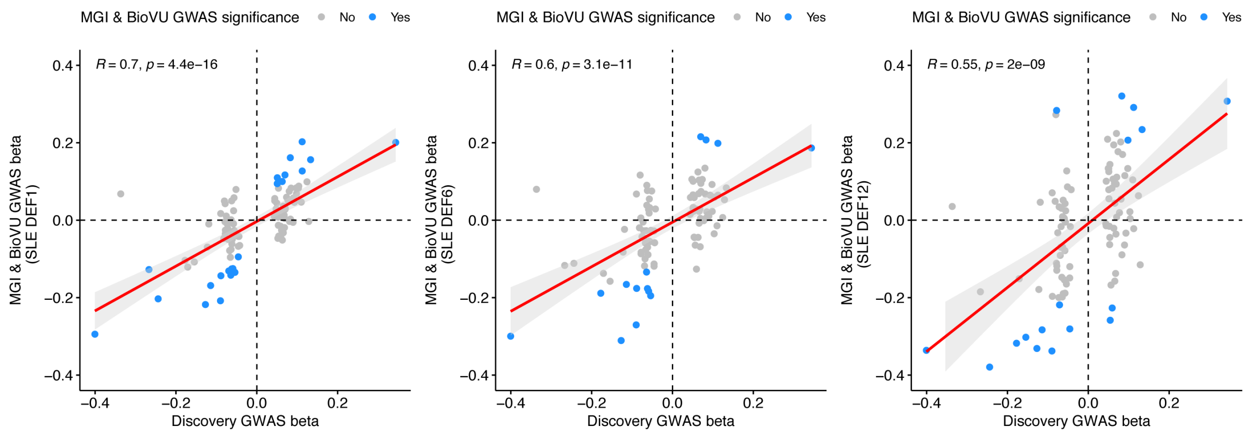

Supplementary Figure 6: Prediction performance of PRS models in MGI and BioVU dataset. We use two metrics to evaluate model performance, including proportion of variance explained on the liability scale (R^2) and area under the receiver operating characteristic curve (AUC). The left panels denote the prediction accuracy of pruning and thresholding method using different P value thresholds. The right panels denote the prediction accuracy of other PRS methods. Error bars represent 95% confidence intervals estimated from bootstrap with 1,000 replicates.

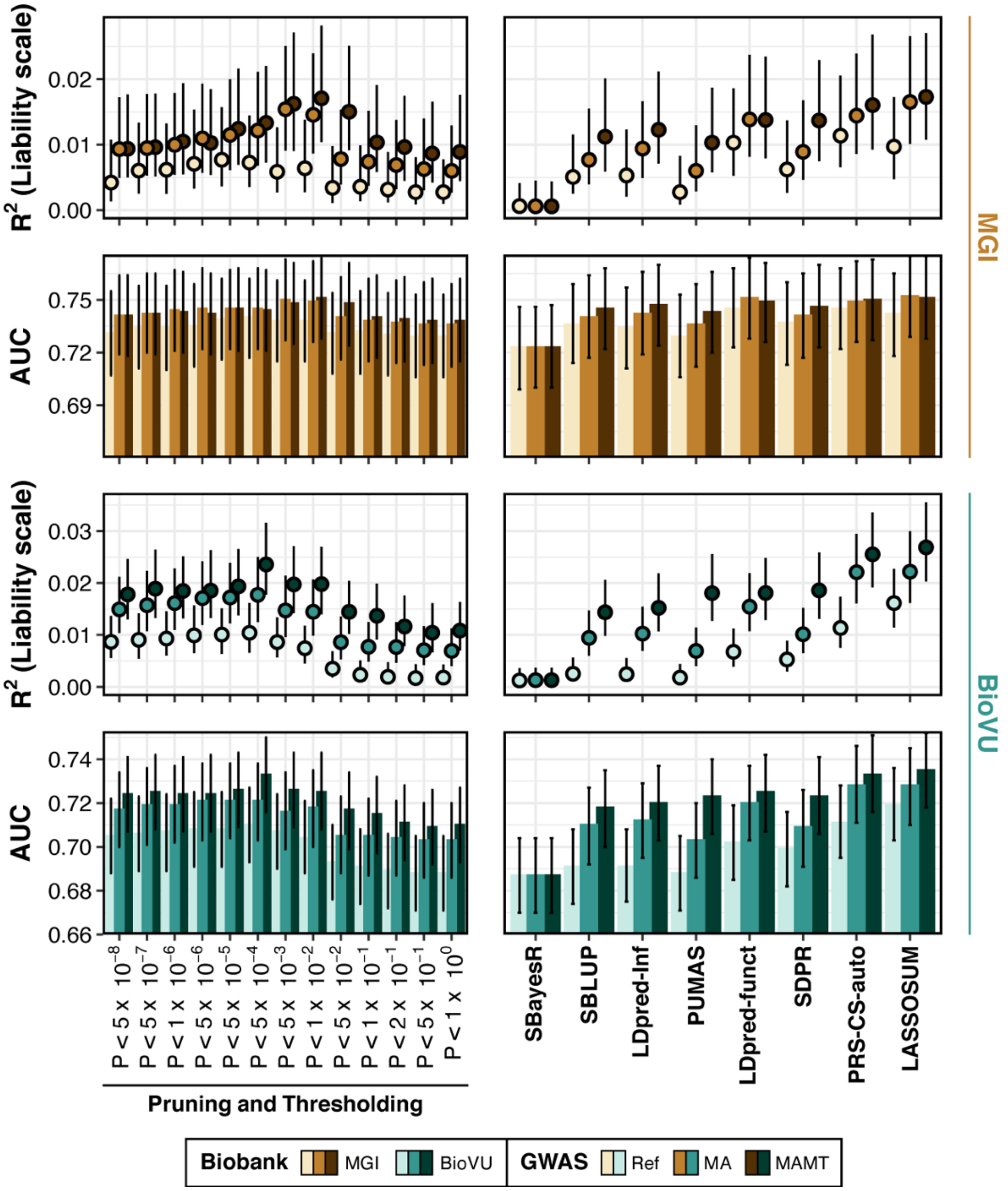

Abbreviations: Ref = reference SLE GWAS data, MA = multi-ancestry fixed effect meta-analysis SLE GWAS data, MAMT = multi-ancestry and multi-trait meta-analysis SLE GWAS data

Supplementary Figure 7: Sensitivity analyses of PRS models in MGI and BioVU dataset. We define SLE cases using three different algorithms according to Barnado et al., 2017. Def12 refers to the most stringent algorithm, while Def1 refers to the least stringent algorithm. Here, we compare different methods using the AUC of ROC. The left panels denote the prediction accuracy of using variants satisfying different P value thresholds for pruning and thresholding method and the right panels denote the prediction accuracy of other PRS methods. Detailed description of different algorithms to identify SLE cases from electronic medical records can be found on **Supplementary Table 4**. A detailed comparison of AUCs between LASSOSUM and other PRS methods via two-sided Delong's test for Def1 can be found in **Supplementary Data 9**.

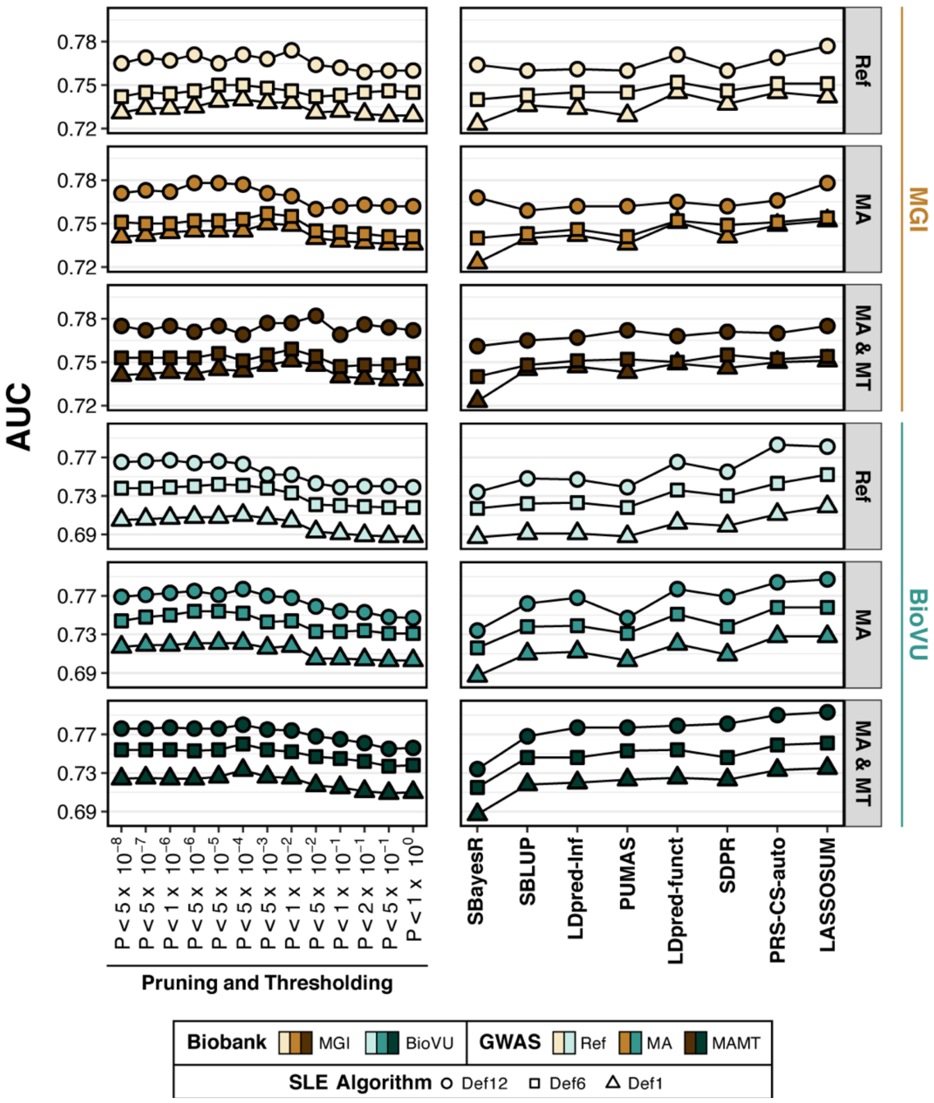

Abbreviations: Ref = reference SLE GWAS data, MA = multi-ancestry fixed effect meta-analysis SLE GWAS data, MAMT = multi-ancestry and multi-trait meta-analysis SLE GWAS data

Supplementary Figure 8: λ_{meta} across 12 SLE cohorts. λ_{meta} is a metric used to detect pairwise cohort heterogeneity and sample overlaps. All λ_{meta} values are close 1, which indicate little evidence of sample overlaps or genetic heterogeneity.

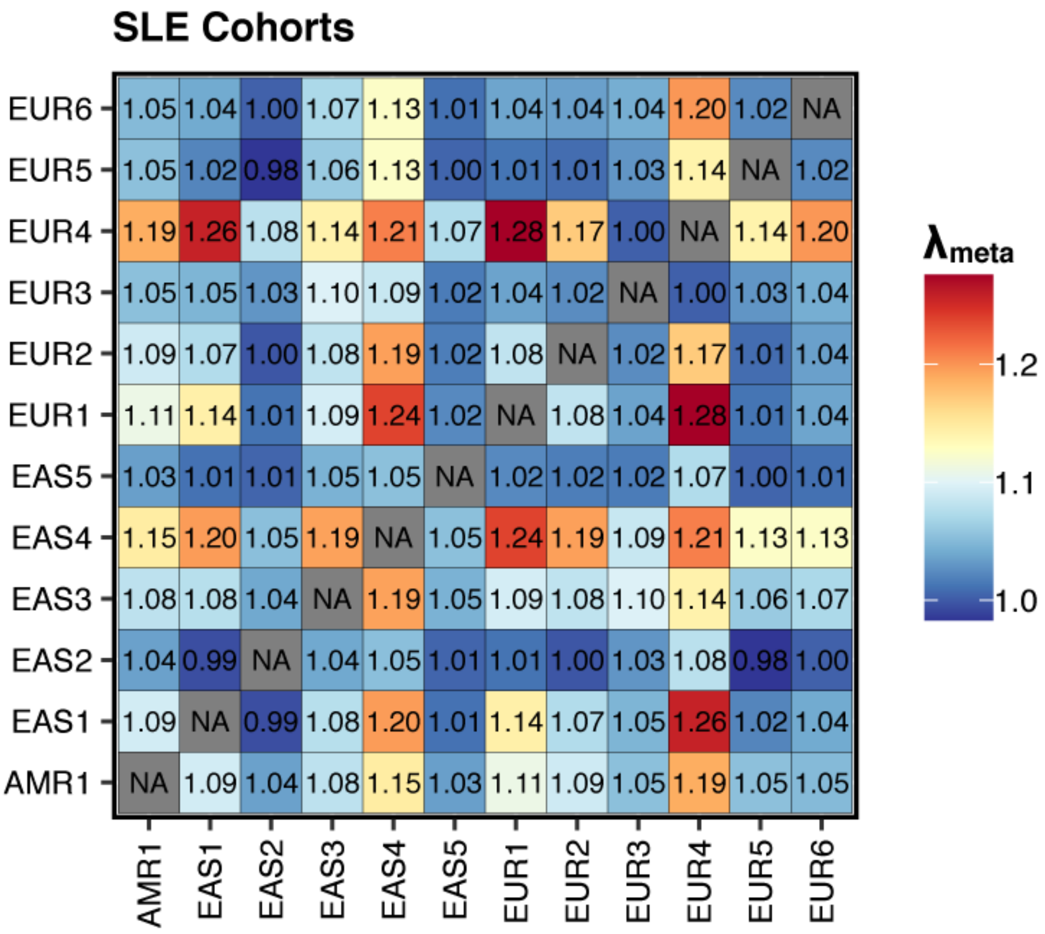

Supplement: Supplementary file 1 — Supplementary Information [file 41467_2023_36306_MOESM1_ESM.pdf]
