# Supplementary material for: Multi-ancestry and multi-trait genome-wide association meta-analyses inform clinical risk prediction for systemic lupus erythematosus
Source: Nat Commun. 2023 Feb 7;14:668. doi: 10.1038/s41467-023-36306-5 (PMC9905560; doi:10.1038/s41467-023-36306-5)
Supplement: Supplementary file 3 — Description of Additional Supplementary Files [file 41467_2023_36306_MOESM3_ESM.pdf]

Description of Additional Supplementary Files

File name: Supplementary Data 1
Description: Studies included in the GWAS meta-analysis across 14 autoimmune diseases. We calculate the effective sample size (N_{eff}) according to the formula $2/(1/N_{cases} + 1/N_{controls})$, which is an informative measurement of sample size for datasets with unbalanced number of cases and controls. Abbreviations: AFR = African, AMR = Admixed American, EAS = East Asian, EUR = European, MID = Middle Eastern, SAS = South Asian, MULTI = Multi-ancestry

File name: Supplementary Data 2
Description: List of 249 sentinel variants from the multi-ancestry and multi-trait SLE GWAS meta-analysis via LD pruning. We use the reference allele as the effect allele, and report the effect size estimates, standard errors, P values, and mapped target gene based on Open Target database. P value associated with each variant is calculated according to the Chi-squared test statistic with 1 degree of freedom.

File name: Supplementary Data 3
Description: Posterior probability of replicability (PPR) for sentinel variants identified from multi-ancestry and multi-trait meta-analysis. In total, we identify 79 known loci and 27 novel loci, of which 74 known loci and 16 novel loci are deemed replicable with PPR > 0.90 via RATES. We arrange the variants by chromosome and genomic location. Two-sided P value associated with each variant is calculated according to the Chi-squared test statistic with 1 degree of freedom.

File name: Supplementary Data 4
Description: Cochran's Q test results for sentinel variants identified from multi-ancestry and multi-trait meta-analysis. In total, 103 of the 106 loci (97%) have two-sided P values ≥ 0.05/106, which fail to reject the null hypothesis of no genetic effect heterogeneity. We arrange the variants by chromosome and genomic location.

File name: Supplementary Data 5
Description: Previously known SLE loci that do not reach genome-wide significance in our multi-ancestry and multi-trait meta-analysis. In total, there are 94 known loci that do not reach genome-wide significance in the meta-analysis. Known loci are retrieved from GWAS catalog and SLE studies included in the meta-analysis. Mapped target gene is based on the Open Target Genetics database. We report the P values of the sentinel variants in each locus in our analyses. We arrange the loci by chromosome and genomic location. Two-sided P value associated with each variant is calculated according to the Chi-squared test statistic with 1 degree of freedom.

File name: Supplementary Data 6
Description: Top TWAS associations at 106 SLE GWAS loci using DGN gene expression prediction models. We define a GWAS locus as a 1 Mb window surrounding each GWAS sentinel variant. Top TWAS associations at 48 SLE GWAS loci reach transcriptome-wide significance threshold (P value $< 2.5 \times 10^{-6}$; Bonferroni threshold for testing 20,000 genes). We label loci without genes in them as NA. Two-sided P value associated with each variant is calculated according to the Chi-squared test statistic with 1 degree of freedom. Two-sided TWAS P value

associated with each gene is calculated based on the TWAS Z score for gene-based association test.

File name: Supplementary Data 7
Description: P values from TWAS associations at 106 SLE GWAS loci using GEUVADIS gene expression prediction models. We define a GWAS locus as a 1 Mb window surrounding each GWAS sentinel variant. Top TWAS associations at 42 SLE GWAS loci reach transcriptome-wide significance threshold (P value < 2.5×10^{-6}; Bonferroni threshold for testing 20,000 genes). We label loci without genes in them as NA. Two-sided P value associated with each variant is calculated according to the Chi-squared test statistic with 1 degree of freedom. Two-sided P value associated with each gene is calculated based on the TWAS Z score for gene-based association test.

File name: Supplementary Data 8
Description: Performance metrics of PRS models in MGI and BioVU. We report true negative (TN), true positive (TP), false negative (FN), false positive (FP), sensitivity, specificity, odd ratio (OR) per standard deviation (SD), OR of developing SLE for individuals with PRS in top 20th vs. bottom 20th percentile, and its 95% confidence interval (OR [95% CI]), and Nagelkerke's R^2 and its 95% confidence interval (NKR2 [95% CI]).

File name: Supplementary Data 9
Description: Comparison of polygenic risk score (PRS) model performance between LASSOSUM and other PRS methods. P-value thresholds used for P+T method are listed in brackets. Here, the comparison is made for each biobank (BioVU or MGI), training dataset (Ref, MA, or MAMT), and PRS method combinations. Here, SLE cases are defined according to Def1 (**Supplementary Table 4**). AUC1 represents area under the receiver operating characteristic curve (AUC) for "Method1", while AUC2 refers to AUC for "Method2". Corresponding 95% confidence interval (95% CI) is estimated from bootstrap with 1,000 replicates. P value is calculated via two-sided Delong's test.
