# Supplementary material for: Multi-ancestry and multi-trait genome-wide association meta-analyses inform clinical risk prediction for systemic lupus erythematosus
Source: Nat Commun. 2023 Feb 7;14:668. doi: 10.1038/s41467-023-36306-5 (PMC9905560; doi:10.1038/s41467-023-36306-5)
Supplement: Supplementary file 13 — Reporting Summary [file 41467_2023_36306_MOESM13_ESM.pdf]

nature portfolio

Corresponding author(s): Dajiang J. Liu, Bibo Jiang, Lam C. Tsoi

Last updated by author(s): Jan 14, 2023

Reporting Summary

Nature Portfolio wishes to improve the reproducibility of the work that we publish. This form provides structure for consistency and transparency in reporting. For further information on Nature Portfolio policies, see our Editorial Policies and the Editorial Policy Checklist.

Statistics

For all statistical analyses, confirm that the following items are present in the figure legend, table legend, main text, or Methods section.

n/a	Confirmed	
☐	☒	The exact sample size (*n*) for each experimental group/condition, given as a discrete number and unit of measurement
☒	☐	A statement on whether measurements were taken from distinct samples or whether the same sample was measured repeatedly
☐	☒	The statistical test(s) used AND whether they are one- or two-sided<br>*Only common tests should be described solely by name; describe more complex techniques in the Methods section.*
☐	☒	A description of all covariates tested
☐	☒	A description of any assumptions or corrections, such as tests of normality and adjustment for multiple comparisons
☐	☒	A full description of the statistical parameters including central tendency (e.g. means) or other basic estimates (e.g. regression coefficient) AND variation (e.g. standard deviation) or associated estimates of uncertainty (e.g. confidence intervals)
☐	☒	For null hypothesis testing, the test statistic (e.g. *F*, *t*, *r*) with confidence intervals, effect sizes, degrees of freedom and *P* value noted<br>*Give P values as exact values whenever suitable.*
☒	☐	For Bayesian analysis, information on the choice of priors and Markov chain Monte Carlo settings
☒	☐	For hierarchical and complex designs, identification of the appropriate level for tests and full reporting of outcomes
☐	☒	Estimates of effect sizes (e.g. Cohen's *d*, Pearson's *r*), indicating how they were calculated

Our web collection on statistics for biologists contains articles on many of the points above.

Software and code

Policy information about availability of computer code

Data collection	No software was used for data collection.
Data analysis	LDSC software version 1.0.1: https://github.com/bulik/ldsc;<br>MTAG software version 1.0.8: https://github.com/JonJala/mtag;<br>Open Target Genetics: https://genetics.opentargets.org;<br>METASOFT software version 2.0.1: http://genetics.cs.ucla.edu/meta;<br>MAMBA software version 1.0.0: https://github.com/dan11mcguire/mamba;<br>BED file processing, bedtools version 2.29.2 https://bedtools.readthedocs.io/en/latest;<br>PUMICE pipeline and implementation version 1.0.0, https://github.com/ckhunsr1/PUMICE (DOI: 10.5281/zenodo.6426359);<br>TESLA: https://github.com/funfunchen/rareGWAMA;<br>CMap version 1.0: https://clue.io;<br>L1000FWD: https://maayanlab.cloud/L1000FWD;<br>SBayesR version 1.0.0: https://github.com/YinLiLin/hibayes;<br>SBLUP version 1.93.2 beta: https://yanglab.westlake.edu.cn/software/gcta;<br>SDPR version 0.9: https://github.com/eldronzhou/SDPR;<br>LDpred-Inf (bigsnpr) version 1.8.1: https://privefl.github.io/bigsnpr/articles/LDpred2.html;<br>LDpred-funct version 1.0.0: https://github.com/carlaml/LDpred-funct;<br>PUMAS version 1.0: https://github.com/qlu-lab/PUMAS;<br>PRS-CS-auto version 1.0.0: https://github.com/getian107/PRScs;<br>LASSOSUM version 0.4.5: https://github.com/tshmak/lassosum;<br>Genotype data processing, PLINK version 1.9 https://www.cog-genomics.org/plink/1.9;<br>Genotype imputation, Michigan Imputation Server https://imputationserver.sph.umich.edu/index.html;<br>Ancestry estimation, ADMIXTURE version 1.3.0 https://bioinformaticshome.com/tools/descriptions/ADMIXTURE.html;

R Project for Statistical Computing version 4.0 https://www.r-project.org;
R packages, tidyr R package version 1.1.2; dplyr R package version 1.0.2; IRanges R package version 2.24.0; GenomicRanges R package version 1.42.0; glmnet R package version 4.0-2; tidyverse R package version 1.3.0; genefilter R package version 1.72.0; caret R package version 6.0-86; ggpubr R package version 0.4.0; ggforce R package version 0.3.2; reshape2 R package version 1.4.4; ggplot2 R package version 3.3.2; RColorBrewer R package version 1.1-2; ggrepel R package version 0.8.2; colortools R package version 0.1.5; qqman R package version 0.1.4; lattice R package version 0.20-38; data.table R package version 1.13.0; matrixStats R package version 0.56.0; stringr R package version 1.4.0; plink2R R package version 1.1; rareGWAMA R package version 0.4; patchwork R package version 1.1.0.9000.

For manuscripts utilizing custom algorithms or software that are central to the research but not yet described in published literature, software must be made available to editors and reviewers. We strongly encourage code deposition in a community repository (e.g. GitHub). See the Nature Portfolio guidelines for submitting code & software for further information.

Data

Policy information about availability of data

All manuscripts must include a data availability statement. This statement should provide the following information, where applicable:

- Accession codes, unique identifiers, or web links for publicly available datasets
- A description of any restrictions on data availability
- For clinical datasets or third party data, please ensure that the statement adheres to our policy

The GWAS summary statistics of the multi-ancestry and multi-trait SLE meta-analysis result generated in this study have been deposited on the Shiny App [https://liugroupstatgen.shinyapps.io/SLEv] for users to download and interactively explore research results. This meta-analysis result was derived via MTAG and METAL from the datasets provided in Supplementary Table 1. When available, we also obtained GWAS data from FinnGen Release 5 website [https://www.finngen.fi/en/access_results], Pan-UK Biobank website [https://pan.ukbb.broadinstitute.org], and BioBank Japan PheWeb [https://pheweb.jp]. A more detailed information of each study can be found in Supplementary Table 1. For TWAS results, we provided TWAS association statistics from two distinct tissues, including DGN (whole blood) and GEUVADIS (lymphoblastoid cell line). We have also linked GWAS variant to its target gene by labeling the eQTL SNP in the gene expression prediction model with the smallest GWAS P value ("top variant" column). DGN data can be requested at https://www.nimhgenetics.org/request-access/how-to-request-access under "Depression Genes and Networks study (D. Levinson, PI)". GEUVADIS data can be accessed at https://www.ebi.ac.uk/arrayexpress/experiments/E-GEUV-1. Gene expression prediction models were created using PUMICE and TWAS association statistics were calculated by TESLA. DICE dataset can be requested through dbGaP accession number phs001703.v1.p1 [https://www.ncbi.nlm.nih.gov/projects/gap/cgi-bin/study.cgi?study_id=phs001703.v1.p1]. B cell dataset from SLE subjects is available from the NCBI Gene Expression Omnibus under accession number GSE118256 [https://www.ncbi.nlm.nih.gov/geo/query/acc.cgi?acc=GSE118256].

Field-specific reporting

Please select the one below that is the best fit for your research. If you are not sure, read the appropriate sections before making your selection.

☒ Life sciences ☐ Behavioural & social sciences ☐ Ecological, evolutionary & environmental sciences

For a reference copy of the document with all sections, see nature.com/documents/nr-reporting-summary-flat.pdf

Life sciences study design

All studies must disclose on these points even when the disclosure is negative.

Sample size	We assembled the largest GWAS summary statistics for SLE and other autoimmune diseases. Our compiled SLE GWAS dataset contains 12 cohorts from three ancestries (East Asian (EAS), N_EAS = 194,435 (5,877 cases and 188,558 controls); European (EUR), N_EUR = 520,311 (14,355 cases and 505,956 controls); Admixed American (AMR), N_AMR = 3,720 (1,393 cases and 2,327 controls)), with a total number of N= 21,625 cases and 696,841 controls. The number of datasets were determined based on the number of publicly available GWAS summary statistics prior to December 2021.
Data exclusions	Non-European samples were excluded from the external datasets (Vanderbilt BioVU and Michigan Genomics Initiative) since the sample sizes of non-European ancestries are too small and inadequate for constructing and assess the accuracy of polygenic risk scores.
Replication	PRS models were externally validated in two independent datasets, including Vanderbilt BioVU (N = 49,707) and Michigan Genomics Initiative (N = 34,702). Additionally, we assess the replicability of association signals using a statistical method MAMBA and its extension to multi-ancestry GWAS meta-analysis, RATES. MAMBA and RATES assess whether a signal is genuine by examining the strength and consistency of association signals between studies using a rigorous statistical model and assign a posterior probability of replicability (PPR). We retain only variants with PPR > .90 in the results.
Randomization	Randomization is not applicable to genetic association studies in case control and population-based biobanks.
Blinding	Blinding is not applicable to genetic association studies in case control and population-based biobanks. No intervention is implemented.

Reporting for specific materials, systems and methods

We require information from authors about some types of materials, experimental systems and methods used in many studies. Here, indicate whether each material, system or method listed is relevant to your study. If you are not sure if a list item applies to your research, read the appropriate section before selecting a response.

Materials & experimental systems

n/a	Involved in the study
☒	☐ Antibodies
☒	☐ Eukaryotic cell lines
☒	☐ Palaeontology and archaeology
☒	☐ Animals and other organisms
☒	☐ Human research participants
☒	☐ Clinical data
☒	☐ Dual use research of concern

Methods

n/a	Involved in the study
☒	☐ ChIP-seq
☒	☐ Flow cytometry
☒	☐ MRI-based neuroimaging
